# Some New Type Sigma Convergent Sequence Spaces and Some New Inequalities

**DOI:** 10.1155/2014/589765

**Published:** 2014-02-20

**Authors:** Kuddusi Kayaduman, Mehmet Şengönül

**Affiliations:** ^1^Department of Mathematics, Faculty of Arts and Sciences, G. Antep University, 27310 Şehitkamil-Gaziantep, Turkey; ^2^Department of Mathematics, Faculty of Arts and Sciences, Nevşehir Hacı Bektaş Veli University, 50300 Nevşehir, Turkey

## Abstract

We have discussed some important problems about the spaces ${\tilde{V}}_{\sigma }$ and ${\tilde{V}}_{0\sigma }$ of Cesàro sigma convergent and Cesàro null sequence.

## 1. Introduction and Preliminaries

In the theory of the sequence spaces, by using the matrix domain of a particular limitation method, so many sequence spaces have been built and published in famous maths journals. By reviewing the literature, one can reach them easily (for instance, see Başar et al. [1], Kirişçi and Başar [2], Şengönül and Başar [3], Altay [4], Mohiuddine and Alotaibi [5], and numerous). As known, the method to obtain a new sequence space by using convergence field of an infinite matrix is an old method in the theory of sequence spaces. But, the study of convergence field of an infinite matrix in the space of *σ*-convergent sequences is quite new. For example, quite recently, Kayaduman and Şengönül introduced the spaces $\tilde{f}$ and ${\tilde{f}}_{0}$ consisting of the sequences *x* = (*x*_*k*_) such that (∑_*j*=0_^*k*^(*x*_*j*_/(*j* + 1))) ∈ *f*, *f*_0_ and gave some important results on those spaces in [6]. Furthermore, in [7], Şengönül and Kayaduman have introduced the spaces $\hat{f}$ and ${\hat{f}}_{0}$ consisting of the sequences *x* = (*x*_*k*_) such that (∑_*j*=0_^*k*^(*r*_*j*_ *x*_*j*_/*R*_*k*_)) ∈ *f*, *f*_0_.

After here, we will pass to the preliminaries for our study. We will denote the space of all real or complex valued sequences by *w* and we will write *ℓ*_*∞*_, *c*, *c*_0_, *ℓ*_1_, *cs*, and *bs* for the spaces of all bounded, convergent, null sequences, absolutely convergent series, convergent series, and bounded series, respectively. Each linear subspace of *w* is called a sequence space. Let *λ* and *μ* be two sequence spaces and *A* = (*a*_*nk*_) be an infinite matrix of real or complex numbers *a*_*nk*_, where *n*, *k* ∈ *ℕ* = {0,1, 2,…}. Then, we can say that *A* defines a matrix mapping from *λ* to *μ*, and we denote it by writing *A* : *λ* → *μ*, if for every sequence *x* = (*x*_*k*_) ∈ *λ*, the sequence *Ax* = {(*Ax*)_*n*_}, that is *A*-transform of *x*, in *μ* where

(1) $$\begin{array}{c} {\left(Ax\right)}_{n}=\underset{k}{\sum }a_{nk}x_{k},\;\left(n\in \mathbb{N}\right). \end{array}$$

For simplicity in notation, here and in what follows, the summation without limits runs from 0 to *∞*. By (*λ* : *μ*), we denote the class of matrices *A* such that *A* : *λ* → *μ*. Thus, *A* ∈ (*λ* : *μ*) if and only if the series on the right side of (1) converges for each *n* ∈ *ℕ* and every *x* ∈ *λ*, we have *Ax* = {(*Ax*)_*n*_}_*n*∈*ℕ*_ ∈ *μ* for all *x* ∈ *λ*. The *matrix domain* *λ*_*A*_ of an infinite matrix *A* in a sequence space *λ* is defined by

(2) $$\begin{array}{c} \lambda_{A}=\left\{x=\left(x_{k}\right)\in w:Ax\in \lambda \right\}. \end{array}$$

If we take *λ* = *c*, then *c*_*A*_ is called *convergence domain* of *A*. We write the limit of *Ax* as lim⁡_*A*_ *x* = lim⁡_*n*→*∞*_∑_*k*_ *a*_*nk*_ *x*_*k*_, and *A* is called regular if lim⁡_*A*_ *x* = lim⁡*x* for each convergent sequence *x*.

The sets *ℓ*_*∞*_ and *c* are Banach spaces with the norm ||*x*_*n*_|| = sup⁡_*n*_ | *x*_*n*_|. Let *σ* be a one-to-one mapping from *ℕ* into itself. A continuous linear functional *ϕ* on *ℓ*_*∞*_ is said to be an invariant mean or a *σ*-mean if and only if

(i) *ϕ*(*x*) ≥ 0 for all sequence *x* = (*x*_*n*_) with nonnegative terms,
(ii) *ϕ*(*e*) = 1, where *e* = (1,1, 1, ...),
(iii) *ϕ*(*x*_*σ*(*k*)_) = *ϕ*(*x*) for all *x* ∈ *ℓ*_*∞*_. Throughout this paper, we consider the mapping *σ* such that *σ* ^*p*^(*k*) ≠ *k* for all positive integers *k* ≥ 0 and *p* ≥ 1, where *σ* ^*p*^(*k*) is the *p*th iterate of *σ* at *k*. Thus, a *σ*-mean extends the limit functional on *c* in the sense that *ϕ*(*x*) = lim⁡*x* for all *x* ∈ *c* (see [8]). Consequently, *c* ⊂ *V*_*σ*_, where *V*_*σ*_ is the set of bounded sequences all of whose *σ*-means are equal; that is,
  (3) Vσ={x∈ℓ∞:lim⁡ptpn(x)=s  uniformly  in  n},
 where
  (4) $$\begin{array}{c} t_{pn}\left(x\right)=\frac{\left(x_{n}+Tx_{n}+\cdots +T^{p}x_{n}\right)}{\left(p+1\right)},\;t_{-1,n}\left(x\right)=0, \end{array}$$
 [9]. We say that a bounded sequence *x* = (*x*_*k*_) is *σ*-convergent if *x* ∈ *V*_*σ*_. In case *σ*(*k*) = *k* + 1, a *σ*-mean is often called a Banach limit and *V*_*σ*_ is reduced to the set of almost sequences, introduced by Lorentz (see [10]). If *x* = (*x*_*n*_), write *Tx* = (*Tx*_*n*_) = (*x*_*σ*(*n*)_). By *Z*, we denote the set of *σ*-convergent sequences with *σ*-limit zero. It is well known [11] that *x* ∈ *ℓ*_*∞*_ if and only if *Tx* − *x* ∈ *Z*.

Let *K* be a subset of *ℕ*. The natural density *δ* of *K* is defined by *δ*(*K*) = lim⁡_*n*→*∞*_(1/*n*) | {*k* ≤ *n* : *k* ∈ *K*}|, where the vertical bars indicate the number of elements in the enclosed set. The sequence *x* = (*x*_*k*_) is said to be statistically convergent to the number *l* if for every *ε*,  *δ*({*k* : |*x*_*k*_ − *l* | ≥*ε*}) = 0 (see [12]). In this case, we write *st*-lim *x* = *l*. We will also write *st* and *st*_0_ to denote the sets of all statistically convergent sequences and statistically null sequences. Let us consider the following functionals defined on *ℓ*_*∞*_:

(5) $$\begin{array}{c} l\left(x\right)=\underset{k\to \infty }{\mathrm{liminf}}\;x_{k},\;\;L\left(x\right)=\underset{k\to \infty }{\mathrm{limsup}}\;x_{k},\\ q_{\sigma }\left(x\right)=\underset{p\to \infty }{\mathrm{limsup}}\;\underset{n\in \mathbb{N}}{\sup}\frac{1}{p+1}\overset{p}{\underset{i=0}{\sum }}x_{\sigma^{i}(n)},\\ L^{*}\left(x\right)=\underset{p\to \infty }{\mathrm{limsup}}\;\underset{n\in \mathbb{N}}{\sup}\frac{1}{p+1}\overset{p}{\underset{i=0}{\sum }}x_{n+i}. \end{array}$$

In [13], the *σ*-core of a real bounded sequence *x* is defined as the closed interval [−*q*_*σ*_(−*x*), *q*_*σ*_(*x*)], and also the inequalities *q*_*σ*_(*Ax*) ≤ *L*(*x*) (*σ*-core of *Ax*⊆*K*-core of *x*), *q*_*σ*_(*Ax*) ≤ *q*_*σ*_(*x*) (*σ*-core of *Ax*⊆*σ*-core of *x*), for all *x* ∈ *ℓ*_*∞*_, have been studied. Here, the Knopp core, in short *K*-core of *x*, is the interval [*l*(*x*), *L*(*x*)] (see [14]). When *σ*(*n*) = *n* + 1, since *q*_*σ*_(*x*) = L*(*x*), *σ*-core of *x* is reduced to the Banach core, in short *B*-core of *x* defined by the interval [−L*(−*x*), L*(*x*)], (see [15]). The concepts of *B*-core and *σ*-core have been studied by many authors [8, 15–19] and Fridy and Orhan [12] have introduced the notions of statistical boundedness, statistical limit superior (or briefly *st*-lim sup⁡), and statistical limit inferior (or briefly *st*-lim inf⁡), defined that the statistical core (or briefly *st*-core) of a statistically bounded sequence is the closed interval [*st*-lim inf⁡*x*, *st*-lim sup⁡*x*], and also determined necessary and sufficient conditions for a matrix *A* to yield *K*-core(*Ax*)⊆*st*-core(*x*) for all *x* ∈ *ℓ*_*∞*_.

In this paper, we define the spaces of Cesàro sigma convergent and Cesàro null sequences and give some interesting theorems.

## 2. Some New Type Sigma Convergent Sequence Spaces

**Definition 1**

A bounded sequence *x* = (*x*_*k*_) is said to be Cesàro sigma convergent to the number *α* if and only if lim⁡_*n*→*∞*_∑_*k*=0_^*n*^∑_*j*=0_^*k*^(*x*_*σ*^*j*^(*p*)_/(*n* + 1)(*k* + 1)) = *α* uniformly in *p*, and the set of all such sequences is denoted with ${\tilde{V}}_{\sigma }$. If *α* = 0, then we write ${\tilde{V}}_{0\sigma }$ instead of ${\tilde{V}}_{\sigma }$; that is,

(6) V~σ={x∈ℓ∞ :lim⁡n→∞∑k=0n ∑j=0kxσj(p)(n+1)(k+1)=α  uniformly  in  p},V~0σ={x∈ℓ∞ :lim⁡n→∞∑k=0n ∑j=0kxσj(p)(n+1)(k+1)=0  uniformly  in  p},

where *α* = *σ*_*C*_-lim*x*, respectively.

**Definition 2**

A bounded sequence *x* = (*x*_*k*_) is said to be Cesàro sigma bounded if and only if sup⁡_*n*_|∑_*k*=0_^*n*^∑_*j*=0_^*k*^(*x*_*σ*^*j*^(*p*)_/(*n* + 1)(*k* + 1))| < *∞*, and the set of all such sequences is denoted with ${\tilde{V}}_{\sigma }^{\infty }$; that is,

(7) $$\begin{array}{c} {\tilde{V}}_{\sigma }^{\infty }=\left\{x\in \ell_{\infty }:\underset{n}{\sup}\left|\overset{n}{\underset{k=0}{\sum }}\;\overset{k}{\underset{j=0}{\sum }}\frac{x_{\sigma^{j}(p)}}{\left(n+1\right)\left(k+1\right)}\right|<\infty \right\}. \end{array}$$

With the notation of (2), we can write ${\tilde{V}}_{\sigma }={\left(V_{\sigma }\right)}_{C}$ and ${\tilde{V}}_{0\sigma }={\left(V_{0\sigma }\right)}_{C}$, where *C* denotes the Cesàro matrix of one order. Define the sequence *y* = (*y*_*n*_), which will be frequently used as the *C*-transform of a sequence *x* = (*x*_*k*_); that is,

(8) $$\begin{array}{c} y_{k}=\overset{k}{\underset{i=0}{\sum }}\frac{x_{i}}{k+1}\;\forall k\in \mathbb{N}. \end{array}$$

Clearly, if we take *σ*(*p*) = *p* + 1, then the spaces ${\tilde{V}}_{\sigma }$ and ${\tilde{V}}_{0\sigma }$ are reduced to the spaces $\tilde{f}$ and $\tilde{f_{0}}$, respectively. Also, we note that the inclusions ${\tilde{V}}_{0\sigma }\subset {\tilde{V}}_{\sigma }\subset {\tilde{V}}_{\sigma }^{\infty }$ hold.

Now, we begin with the following theorem.

**Theorem 3**

The sequence spaces ${\tilde{V}}_{\sigma }$ and ${\tilde{V}}_{0\sigma }$ are linearly isomorphic to the spaces *V*_*σ*_ and *V*_0*σ*_, respectively; that is, ${\tilde{V}}_{\sigma }≅V_{\sigma }$ and ${\tilde{V}}_{0\sigma }≅V_{0\sigma }$.

**Proof**

We consider only the spaces ${\tilde{V}}_{\sigma }$ and *V*_*σ*_. In order to prove the fact ${\tilde{V}}_{\sigma }≅V_{\sigma }$, we should show the existence of a linear bijection between the spaces ${\tilde{V}}_{\sigma }$ and *V*_*σ*_. Consider the transformation of *C* defined with the notation of (8) from ${\tilde{V}}_{\sigma }$ to *V*_*σ*_ by *x* ↦ *y* = *Cx*. The linearity of *C* is clear. Further, it is trivial that *x* = *θ* = (0,0, ...) whenever *Cx* = *θ* and hence *C* is injective. Let *y* ∈ *V*_*σ*_ and define the sequence *x* by

(9) $$\begin{array}{c} x_{\sigma^{i}(p)}=\left(i+1\right)y_{\sigma^{i}(p)}-iy_{\sigma^{i-1}(p)},\;\left(i\in \mathbb{N}\right). \end{array}$$

Then, we have

(10) lim⁡n→∞∑j=0n ∑i=0jxσi(p)(n+1)(j+1)  =lim⁡n→∞∑j=0n ∑i=0j(i+1)yσi(p)−iyσi−1(p)(n+1)(j+1)  =lim⁡n→∞∑j=0nyσj(p)n+1  uniformly  in  p,

which shows that $x\in {\tilde{V}}_{\sigma }$. Consequently, we see that *C* is surjective. Hence, *C* is linear bijection which therefore shows that the spaces ${\tilde{V}}_{\sigma }$ and *V*_*σ*_ are linearly isomorphic, as desired. This completes the proof. The fact that the spaces ${\tilde{V}}_{0\sigma }$ and *V*_0*σ*_ are linearly isomorphic can also be proved by the similar way, so we omit it.

**Remark 4 (see [20])**

The spaces *V*_*σ*_ and *V*_0*σ*_ are BK-spaces with the norm ||·||_*ℓ*_*∞*__.

**Theorem 5**

The sets ${\tilde{V}}_{\sigma }^{\infty }$, ${\tilde{V}}_{\sigma }$, and ${\tilde{V}}_{0\sigma }$ are linear spaces with the coordinatewise addition and scalar multiplication which are BK-spaces with the norm

(11) $$\begin{array}{c} {||x||}_{{\tilde{V}}_{\sigma }}=\underset{n}{\sup}\left|\overset{n}{\underset{j=0}{\sum }}\;\overset{j}{\underset{i=0}{\sum }}\frac{x_{\sigma^{i}(p)}}{\left(n+1\right)\left(j+1\right)}\right|. \end{array}$$

**Proof**

The proof of first part of the theorem is easy. We will prove second part of the theorem. Since (8) holds, the spaces *V*_*σ*_ and *V*_0*σ*_ are BK-spaces with the norm ||·||_*∞*_, (see Remark 4), the matrix *C* is normal and Theorem 4.3.2 of Wilansky [21] gives the fact that the spaces ${\tilde{V}}_{\sigma }$ and ${\tilde{V}}_{0\sigma }$ are BK-spaces.

It is known that, if *λ* and *μ* are normed spaces, then the set *G*(*T*) = {(*x*, *T*(*x*)) : *x* ∈ *λ*} is subspace of *λ* × *μ* and the set *G*(*T*) is normed space with the norm

(12) $$\begin{array}{c} {||\left(x,T\left(x\right)\right)||}_{G(T)}={||x||}_{\lambda }+{||y||}_{\mu }, \end{array}$$

where *T* is a linear transformation from *λ* to *μ*.

**Theorem 6**

The graph *G*(*C*) of the transformation *C* is closed subspace in ${\tilde{V}}_{\sigma }\times V_{\sigma }$.

**Proof**

We know that the spaces ${\tilde{V}}_{\sigma }$ and *V*_*σ*_ are Banach spaces (see, Theorem 5 and Remark 4) and the transformation *C* is linear and continuous from ${\tilde{V}}_{\sigma }$ to *V*_*σ*_. Let us suppose that the sequence (*x*_*n*_, *C*(*x*_*n*_)) is convergent to (*x*, *y*) in *G*(*C*) for $x\in {\tilde{V}}_{\sigma }$ and *y* ∈ *V*_*σ*_. With this supposition, and from the equality

(13) $$\begin{array}{c} {||\left(x_{n},C\left(x_{n}\right)\right)-\left(x,y\right)||}_{G(C)}={||x_{n}-x||}_{{\tilde{V}}_{\sigma }}+{||C\left(x_{n}\right)-y||}_{V_{\sigma }}, \end{array}$$

we see that *x*_*n*_ → *x* and *C*(*x*_*n*_) → *y* as *n* → *∞*. Also, since *C* is continuous and from the definition of the sequential continuous, we obtain that *Cx* = *y* and this completes the proof.

## 3. Some Matrix Mappings Related to the Space ${\tilde{V}}_{\sigma }$

Following Başar [22], we start with giving short knowledge on the dual summability methods of the new type.

Let us suppose that the infinite matrices *A* = (*a*_*nk*_) and *B* = (*b*_*nk*_) map the sequences *x* = (*x*_*k*_) and *y* = (*y*_*k*_) which are connected by the relation (8) to the sequences *z* = (*z*_*n*_) and *t* = (*t*_*n*_), respectively; that is,

(14) $$\begin{array}{c} z_{n}={\left(Ax\right)}_{n}=\underset{k}{\sum }a_{nk}x_{k},\;\left(n\in \mathbb{N}\right),\\ t_{n}={\left(By\right)}_{n}=\underset{k}{\sum }b_{nk}y_{k},\;\left(n\in \mathbb{N}\right). \end{array}$$

It is clear here that the method *B* is applied to the *C*-transform of the sequence *x* = (*x*_*k*_) while the method *A* is directly applied to the entries of the sequence *x* = (*x*_*k*_). So, the methods *A* and *B* are essentially different. Let us assume that the matrix product *BC* exists which is a much weaker assumption than the conditions on the matrix *B* belonging to any matrix class, in general. The methods *A* and *B* in (14) are called * dual summability methods of the new type* if *z*_*n*_ reduces to *t*_*n*_ (or *t*_*n*_ reduces to *z*_*n*_) under the application of formal summation by parts. This leads us to the fact that *BC* exists and is equal to *A* and (*BC*)*x* = *B*(*Cx*) formally holds, if one side exists. This statement is equivalent to the following relation between the entries of the matrices *A* = (*a*_*nk*_) and *B* = (*b*_*nk*_):

(15) ank:=∑j=k∞bnjj+1 orbnk:=(k+1)(ank−an,k+1)=(k+1)Δank,

for all *n*, *k* ∈ *ℕ*.

**Lemma 7 (see [9])**

*A* ∈ (*ℓ*_*∞*_, *V*_*σ*_), if and only if

(16) $$\begin{array}{c} ||A||=\underset{n}{\sup}\underset{k}{\sum }\left|a_{nk}\right|<\infty , \end{array}$$

(17) $$\begin{array}{c} \sigma \text{-}\underset{}{\lim}a_{nk}=\alpha_{k}\;for\;\;each\;\;k, \end{array}$$

(18) $$\begin{array}{c} \underset{p}{\lim}\underset{k}{\sum }\frac{1}{p+1}\left|\overset{p}{\underset{i=0}{\sum }}\left(a_{\sigma^{i}(n),k}-\alpha_{k}\right)\right|\;\;uniformly\;\;in\;\;n. \end{array}$$

**Lemma 8 (see [9])**

*A* ∈ (*c*, *V*_*σ*_), if and only if (16) and (17) hold, and

(19) $$\begin{array}{c} \sigma \text{-}\underset{n}{\lim}\underset{k}{\sum }a_{nk}=\alpha . \end{array}$$

**Lemma 9**

*A* ∈ (*V*_*σ*_, *V*_*σ*_), if and only if (16) and (17) hold, and

(20) $$\begin{array}{c} A\left(T-I\right)\in \left(m,V_{\sigma }\right), \end{array}$$

where *I* is identity matrix.

Now, we give the following theorem concerning to the dual matrices of the new type.

**Theorem 10**

Suppose that the entries of the infinite matrices *D* = (*d*_*nk*_) and *E* = (*e*_*nk*_) are connected with the relation

(21) $$\begin{array}{c} e_{nk}=\overset{n}{\underset{j=0}{\sum }}\frac{d_{jk}}{n+1},\;\left(n,k\in \mathbb{N}\right), \end{array}$$

and let *μ* be any given sequence space. Then, $D\in \left(\mu :{\tilde{V}}_{\sigma }\right)$, if and only if *E* ∈ (*μ* : *V*_*σ*_).

**Proof**

Let *x* = (*x*_*k*_) ∈ *μ* and consider the following equality with (21):

(22) $$\begin{array}{c} \overset{n}{\underset{j=0}{\sum }}\;‍\overset{m}{\underset{k=0}{\sum }}\frac{d_{jk}x_{\sigma^{k}(p)}}{n+1}=\overset{m}{\underset{k=0}{\sum }}e_{nk}x_{\sigma^{k}(p)};\;\left(m,n,k\in \mathbb{N}\right), \end{array}$$

which yields as *m* → *∞* that $Dx\in {\tilde{V}}_{\sigma }$, whenever *x* ∈ *μ*, if and if only *Ex* ∈ *V*_*σ*_, whenever *x* ∈ *μ*. This step completes the proof.

If we take *σ*(*p*) = *p* + 1, then Theorem 10 is reduced to Theorem 5.2 of Kayaduman and Şengönül, [6].

Now, right here, we have stated two theorem which are natural consequences of the Lemma 7, Lemma 8, and Theorem 10.

**Theorem 11**

Let *A* = (*a*_*nk*_) be an infinite matrix real or complex numbers. Then, $A=\left(a_{nk}\right)\in \left(V_{\sigma }:\tilde{V_{\sigma }}\right)$ if and only if

1. sup⁡_*n*∈*ℕ*_∑_*k*_|∑_*j*=*k*_^*∞*^(*a*_*nj*_/(*j* + 1))| < *∞*,  
2. *σ*-lim⁡_*n*→*∞*_∑_*k*_∑_*j*=*k*_^*∞*^(*a*_*nj*_/(*j* + 1)) = *α*,  
3. $A\left(T-I\right)\in \left(\ell_{\infty }:\tilde{V_{\sigma }}\right)$,

where *I* is the identity matrix.

**Theorem 12**

Let *A* = (*a*_*nk*_) be an infinite matrix real or complex numbers. Then, $A=\left(a_{nk}\right)\in \left(\ell_{\infty }:{\tilde{V}}_{\sigma }\right)$ if and only if

(4) sup⁡_*n*∈*ℕ*_∑_*k*_|∑_*j*=*k*_^*∞*^(*a*_*nj*_/(*j* + 1))| < *∞*,
(5) *σ*-lim⁡_*n*→*∞*_∑_*j*=*k*_^*∞*^(*a*_*nj*_/(*j* + 1)) = *α*_*k*_, exists for each fixed *k* ∈ *ℕ*,
(6) lim⁡_*m*→*∞*_∑_*k*_|∑_*i*=0_^*m*^∑_*j*=*k*_^*∞*^(*a*_*σ*^*i*^(*n*),*j*_/(*m* + 1)(*j* + 1)) − *α*_*k*_| = 0, uniformly in *n*.

**Proof**

The proof is clear from Theorem 10 and Lemma 7.

**Theorem 13**

Let *A* = (*a*_*nk*_) be an infinite matrix real or complex numbers. Then, $A=\left(a_{nk}\right)\in \left(c:{\tilde{V}}_{\sigma }\right)$ if and only if

(7) sup⁡_*n*∈*ℕ*_∑_*k*_|∑_*j*=*k*_^*∞*^(*a*_*nj*_/(*j* + 1))| < *∞*,
(8) *σ*-lim⁡_*n*→*∞*_∑_*j*=*k*_^*∞*^(*a*_*nj*_/(*j* + 1)) = *α*_*k*_ exists for each fixed *k* ∈ *ℕ*,
(9) *σ*-lim⁡_*n*→*∞*_∑_*k*_∑_*j*=*k*_^*∞*^(*a*_*nj*_/(*j* + 1)) = *α*.

**Proof**

The proof is clear from Theorem 10 and Lemma 8.

Furthermore, from the Lemma 3.2 of [23], we have the following proposition.

**Proposition 14**

Suppose that the entries of the infinite matrices *A* = (*a*_*nk*_) and *B* = (*b*_*nk*_) are connected with the relation

(23) $$\begin{array}{c} a_{nk}:=\overset{\infty }{\underset{j=k}{\sum }}\frac{b_{nj}}{j+1},\;\left(n,k\in \mathbb{N}\right), \end{array}$$

and let *μ* be any given sequence space. Then, if $A\in \left({\tilde{V}}_{\sigma }:\mu \right)$, then

(10) *α*_*k*_ = lim⁡_*n*_∑_*j*=*k*_^*∞*^(*b*_*nj*_/(*j* + 1))* exists for every k* ∈ *ℕ*,
(11) sup⁡_*n*_(∑_*k*_ | ∑_*j*=*k*_^*∞*^(*b*_*nj*_/(*j* + 1)) − *α*_*k*_|) < *∞*,
(12) *α* = ∑_*k*_ | *α*_*k*_ | <*∞*.

## 4. Some Inequalities

In this section, we use matrices classes $\left(\ell_{\infty }:{\tilde{V}}_{\sigma }\right)$, $\left(c:{\tilde{V}}_{\sigma }\right)$, and $\left(V_{\sigma }:\tilde{V_{\sigma }}\right)$ to show the following inequalities: ${\tilde{q}}_{\sigma }\left(Ax\right)\leq L\left(x\right)$, ${\tilde{q}}_{\sigma }\left(Ax\right)\leq q_{\sigma }\left(x\right)$, and ${\tilde{q}}_{\sigma }\left(Ax\right)\leq \beta \left(x\right)$, which are analogues of Knopp's core theorem.

**Definition 15**

Let *x* ∈ *ℓ*_*∞*_. Then, *σ*_*C*_-core of *x* is defined by the closed interval $\left[-{\tilde{q}}_{\sigma }\left(-x\right),{\tilde{q}}_{\sigma }\left(x\right)\right]$, where

(24) $$\begin{array}{c} {\tilde{q}}_{\sigma }\left(x\right)=\underset{n\to \infty }{\mathrm{limsup}}\;\underset{p\in \mathbb{N}}{\sup}\overset{n}{\underset{k=0}{\sum }}\;\overset{k}{\underset{j=0}{\sum }}\frac{x_{\sigma^{j}(p)}}{\left(n+1\right)\left(k+1\right)}. \end{array}$$

Therefore, it is easy to see that *σ*_*C*_-core of *x* is *α* if and only if *σ*_*C*_-lim*x* = *α*.

We need the following lemma due to Das [17] for the proof of next theorem.

**Lemma 16**

Let ||*C*|| = ||*c*_*ni*_(*p*)|| < *∞* and let lim⁡_*n*→*∞*_sup⁡_*p*∈*ℕ*_ | *c*_*ni*_(*p*) | = 0. Then, there is a *y* = (*y*_*i*_) ∈ *ℓ*_*∞*_ such that ||*y*|| ≤ 1 and

(25) $$\begin{array}{c} \underset{n\to \infty }{\mathrm{limsup}}\;\underset{p\in \mathbb{N}}{\sup}\underset{i}{\sum }c_{ni}\left(p\right)y_{i}=\underset{n\to \infty }{\mathrm{limsup}}\;\underset{p\in \mathbb{N}}{\sup}\underset{i}{\sum }\left|c_{ni}\left(p\right)\right|. \end{array}$$

**Lemma 17**

Let *P* and *Q* be sublinear functionals on a linear space *X*. Then, {*X*, *P*}⊂{*X*, *Q*} if and only if *P*(*x*) ≤ *Q*(*x*) for all *x* ∈ *X*.

**Theorem 18**

${\tilde{q}}_{\sigma }\left(Ax\right)\leq L\left(x\right)$ for all *x* ∈ *ℓ*_*∞*_ if and only if $A\in {\left(c:{\tilde{V}}_{\sigma }\right)}_{reg}$ and

(26) $$\begin{array}{c} \underset{n\to \infty }{\lim}\underset{i}{\sum }\overset{-}{a}\left(n,i,p\right)=1\;\;uniformly\;\;in\;\;p, \end{array}$$

where $\overset{-}{a}\left(n,i,p\right)=\sum_{k=0}^{n}\sum_{j=0}^{k}\left(a_{\sigma^{j}(p),i}/\left(n+1\right)\left(k+1\right)\right)$ for all *n*, *i*, *p* ∈ *ℕ*.

**Proof**

Consider the following.

*Sufficiency.* Since *x* ∈ *ℓ*_*∞*_, it is known that for any given *ϵ* > 0, there exists a positive integer *i*_0_ such that *x*_*i*_ ≤ *L*(*x*) + *ϵ* whenever *i* ≥ *i*_0_. Now, let us write

(27) ∑ia−(n,i,p)xi=∑i<i0a−(n,i,p)xi+∑i≥i0a−(n,i,p)+xi−∑i≥i0a−(n,i,p)−xi.

Then, by hypothesis, the first and the last sums on the right-hand side of (27) tend to zero, as *n* → *∞*, uniformly in *p*. Therefore, we obtain by applying the operator limsup⁡_*n*→*∞*_sup⁡_*p*∈*ℕ*_ to the equality (27) that

(28) $$\begin{array}{c} {\tilde{q}}_{\sigma }\left(Ax\right)\leq \left[L\left(x\right)+\epsilon \right]\underset{n\to \infty }{\mathrm{limsup}}\;\underset{p\in \mathbb{N}}{\sup}\underset{i}{\sum }\overset{-}{a}\left(n,i,p\right). \end{array}$$

Since (26) holds, we have ${\tilde{q}}_{\sigma }\left(Ax\right)\leq L\left(x\right)$.

*Necessity.* We observe by inserting −*x* = (−*x*_*i*_) in place of *x* = (*x*_*i*_) in the inequality ${\tilde{q}}_{\sigma }\left(Ax\right)\leq L\left(x\right)$ that

(29) $$\begin{array}{c} \ell \left(x\right)\leq -{\tilde{q}}_{\sigma }\left(-Ax\right)\leq {\tilde{q}}_{\sigma }\left(Ax\right)\leq L\left(x\right). \end{array}$$

If *x* ∈ *c*, then *ℓ*(*x*) = *L*(*x*) = lim⁡*x* and so $-{\tilde{q}}_{\sigma }\left(-Ax\right)={\tilde{q}}_{\sigma }\left(Ax\right)$ which means that *σ*_*C*_-lim*Ax* = lim⁡ *x*. Hence, $A\in {\left(c:{\tilde{V}}_{\sigma }\right)}_{\text{reg}}$. It is clear that

(30) $$\begin{array}{c} \underset{n\to \infty }{\mathrm{liminf}}\underset{i}{\sum }\left|\overset{-}{a}\left(n,i,p\right)\right|\geq \underset{n\to \infty }{\mathrm{liminf}}\underset{i}{\sum }\overset{-}{a}\left(n,i,p\right)=1, \end{array}$$

uniformly in *p*. On the other hand, if we choose the sequence of matrices $𝒜=\{{\overset{-}{a}}_{in}\left(p\right)\}$ defined by ${\overset{-}{a}}_{in}\left(p\right)=\overset{-}{a}\left(n,i,p\right)$, for all *n*, *i*, *p* ∈ *ℕ*, then clearly *𝒜* satisfies the conditions of Lemma 16. So, there exists a *y* = (*y*_*i*_) ∈ *ℓ*_*∞*_ with ||*y*|| ≤ 1 and

(31) $$\begin{array}{c} \underset{n\to \infty }{\mathrm{limsup}}\;\underset{p\in \mathbb{N}}{\sup}\underset{i}{\sum }\left|\overset{-}{a}\left(n,i,p\right)\right|=\underset{n\to \infty }{\mathrm{limsup}}\;\underset{p\in \mathbb{N}}{\sup}\underset{i}{\sum }\overset{-}{a}\left(n,i,p\right)y_{i}. \end{array}$$

Thus, we derive by inserting this *y* in the hypothesis that

(32) $$\begin{array}{c} \underset{n\to \infty }{\mathrm{limsup}}\;\underset{p\in \mathbb{N}}{\sup}\underset{i}{\sum }\left|\overset{-}{a}\left(n,i,p\right)\right|={\tilde{q}}_{\sigma }\left(Ay\right)\leq L\left(y\right)\leq ||y||\leq 1. \end{array}$$

Combining this result by the fact in (30), we obtain the required condition.

In the special case *σ*(*p*) = *p* + 1, we also have the following.

**Theorem 19 (see [6])**

*B*_*C*_-*core*(*Ax*)⊆*K*-*core*(*x*) for all *x* ∈ *ℓ*_*∞*_ if and only if $A\in {\left(c:\tilde{f}\right)}_{reg}$ and

(33) $$\begin{array}{c} \underset{n\to \infty }{\lim}\;\underset{p\in \mathbb{N}}{\sup}\underset{i}{\sum }\frac{1}{n+1}\left|\overset{n}{\underset{k=0}{\sum }}\frac{1}{k+1}\overset{k}{\underset{j=0}{\sum }}a_{j+p,i}\right|=1. \end{array}$$

**Corollary 20**

${\tilde{q}}_{\sigma }\left(Ax\right)\leq q_{\sigma }\left(x\right)$ for all *x* ∈ *ℓ*_*∞*_ if and only if $A\in {\left(V_{\sigma }:{\tilde{V}}_{\sigma }\right)}_{reg}$ and (26) holds.

**Proof**

Consider the following.

*Necessity.* By the similar way used in the proof of necessity of Theorem 18, one can see that $A\in {\left(V_{\sigma }:{\tilde{V}}_{\sigma }\right)}_{\text{reg}}$. On the other hand, since *q*_*σ*_(*x*) ≤ *L*(*x*) for any sequence *x*, condition (26) follows from Theorem 18.

*Sufficiency.* Suppose that ${\left(V_{\sigma }:{\tilde{V}}_{\sigma }\right)}_{\text{reg}}$ and (26) hold. Since ${\left(V_{\sigma }:{\tilde{V}}_{\sigma }\right)}_{\text{reg}}$ implies that $A\in {\left(c:{\tilde{V}}_{\sigma }\right)}_{\text{reg}}$, from Theorem 18,

(34) $$\begin{array}{c} u\left(x\right)=\underset{z\in V_{0\sigma }}{\inf}{\tilde{q}}_{\sigma }\left(Ax+z\right)\leq \underset{z\in V_{0\sigma }}{\inf}L\left(x+z\right)=v\left(x\right). \end{array}$$

On the other hand, since ${\tilde{q}}_{\sigma }\left(Az\right)=-{\tilde{q}}_{\sigma }\left(-Az\right)=0$ for *z* ∈ *V*_0*σ*_, we have

(35) $$\begin{array}{c} u\left(x\right)\geq \underset{z\in V_{0\sigma }}{\inf}\left\{{\tilde{q}}_{\sigma }\left(Ax\right)+\left[-{\tilde{q}}_{\sigma }\left(-Az\right)\right]\right\}={\tilde{q}}_{\sigma }\left(Ax\right). \end{array}$$

Now, combining (34) and (35), we obtain ${\tilde{q}}_{\sigma }\left(Ax\right)\leq v\left(x\right)$ for all *x* ∈ *ℓ*_*∞*_. Since *v*(*x*) = *q*_*σ*_(*x*) [13], the proof is completed.

In the special case *σ*(*p*) = *p* + 1, we also have the following theorem.

**Theorem 21 (see [6])**

One can see that *B*_*C*_-*core*(*Ax*)⊆*B*-*core*(*x*) for all *x* ∈ *ℓ*_*∞*_ if and only if $A\in {\left(f:\tilde{f}\right)}_{reg}$ and (33) holds.

**Corollary 22**

$A\in {\left(st\cap \ell_{\infty }:{\tilde{V}}_{\sigma }\right)}_{reg}$ if and only if $A\in {\left(c:{\tilde{V}}_{\sigma }\right)}_{reg}$ and

(36) $$\begin{array}{c} \underset{n\to \infty }{\lim}\underset{i\in E}{\sum }\frac{1}{n+1}\left|\overset{n}{\underset{k=0}{\sum }}\;\overset{k}{\underset{j=0}{\sum }}\frac{a_{\sigma^{j}(p),i}}{\left(k+1\right)}\right|=0\;\;uniformly\;\;in\;\;p, \end{array}$$

for every *E*⊆*ℕ* with natural density zero.

**Proof**

Let $A\in {\left(st\cap \ell_{\infty }:{\tilde{V}}_{\sigma }\right)}_{\text{reg}}$. Then, $A\in {\left(c:{\tilde{V}}_{\sigma }\right)}_{\text{reg}}$ immediately follows from the fact that *c* ⊂ *st*∩*ℓ*_*∞*_. Now, define a sequence *t* = (*t*_*i*_) via *x* ∈ *ℓ*_*∞*_ as

(37) $$\begin{array}{c} t_{i}=\{\begin{array}{cc} x_{i}, & i\in E,\\ 0, & i\notin E, \end{array} \end{array}$$

where *E* is any subset of *ℕ* with *δ*(*E*) = 0. Then, *st*-lim*t*_*i*_ = 0 and *t* ∈ *st*_0_, so we have $At\in {\tilde{V}}_{0\sigma }$. On the other hand, since (*At*)_*n*_ = ∑_*i*∈*E*_ *a*_*ni*_ *t*_*i*_, the matrix *B* = (*b*_*ni*_), defined by

(38) $$\begin{array}{c} b_{ni}=\{\begin{array}{cc} a_{ni}, & i\in E,\\ 0, & i\notin E, \end{array} \end{array}$$

for all *n*, must belong to the class $\left(\ell_{\infty }:{\tilde{V}}_{0\sigma }\right)$. Hence, the necessity of (36) follows from (4) part of Theorem 12. Conversely, suppose that $A\in {\left(c:{\tilde{V}}_{\sigma }\right)}_{\text{reg}}$ and (36) holds. Let *x* ∈ *st*∩*ℓ*_*∞*_ and let *st*-lim *x* = *ℓ*. Write *E* = {*i* : |*x*_*i*_ − *ℓ* | ≥*ε*} for any given *ε* > 0, so that *δ*(*E*) = 0. Since $A\in {\left(c,{\tilde{V}}_{\sigma }\right)}_{\text{reg}}$ and *σ*_*C*_-lim⁡_*n*→*∞*_∑_*i*_ *a*_*ni*_ = 1, we have

(39) σC-lim(Ax)=σC-lim(∑iani(xi−ℓ)+ℓ∑iani)=σC-lim(∑iani(xi−ℓ)+ℓ)=lim⁡n→∞sup⁡p∈ℕ∑i ∑k=0n ∑j=0kaσj(p),i(n+1)(k+1)×(xi−ℓ)+ℓ.

On the other hand, since

(40) |∑i ∑k=0n ∑j=0kaσj(p),i(n+1)(k+1)(xi−ℓ)|  ≤||x||∞∑i∈E1n+1|∑k=0n ∑j=0kaσj(p),i(k+1)|+ε||A||,

the condition (36) implies that

(41) lim⁡n→∞∑i ∑k=0n ∑j=0kaσj(p),i(n+1)(k+1)(xk−ℓ)=0uniformly  in  p.

Hence, *σ*_*C*_-lim(*Ax*) = *st*-lim *x*; that is, $A\in {\left(st\cap m:{\tilde{V}}_{\sigma }\right)}_{\text{reg}}$, which completes the proof.

In the special case *σ*(*p*) = *p* + 1, we also have the following theorem.

**Theorem 23 (see [6])**

*Consider that* $A\in {\left(S\cap \ell_{\infty }:\tilde{f}\right)}_{reg}$ if and only if $A\in {\left(c:\tilde{f}\right)}_{reg}$ and

(42) $$\begin{array}{c} \underset{n\to \infty }{\lim}\underset{i\in E}{\sum }\frac{1}{n+1}\left|\overset{n}{\underset{k=0}{\sum }}\frac{1}{k+1}\overset{k}{\underset{j=0}{\sum }}a_{j+p,i}\right|=0\;uniformly\;\;in\;\;p, \end{array}$$

for every *E*⊆*ℕ* with natural density zero.

**Corollary 24**

Consider that ${\tilde{q}}_{\sigma }\left(Ax\right)\leq \beta \left(x\right)$ for all *x* ∈ *ℓ*_*∞*_ if and only if $A\in {\left(st\cap \ell_{\infty }:{\tilde{V}}_{\sigma }\right)}_{reg}$ and (26) holds.

**Proof**

Consider the following.

*Necessity*. Firstly, assume that ${\tilde{q}}_{\sigma }\left(Ax\right)\leq \beta \left(x\right)$ for all *x* ∈ *ℓ*_*∞*_ where *β*(*x*) = *st*-lim sup⁡ *x*. Hence, since *β*(*x*) = *st*-lim sup⁡ *x* ≤ *L*(*x*) for all *x* ∈ *ℓ*_*∞*_ (see [12]), we have (26) from Theorem 18. Furthermore, one can also easily see that $-\beta \left(-x\right)\leq -{\tilde{q}}_{\sigma }\left(-Ax\right)\leq {\tilde{q}}_{\sigma }\left(Ax\right)\leq \beta \left(x\right)$; that is, $st\text{-lim}\;\inf\;x\leq -{\tilde{q}}_{\sigma }\left(-Ax\right)\leq {\tilde{q}}_{\sigma }\left(Ax\right)\leq st\text{-lim}\;\sup\;x$. If *x* ∈ *st*∩*ℓ*_*∞*_, then *st*-lim inf⁡ *x* = *st*-lim sup⁡ *x* = *st*-lim *x*. Thus, the last inequality implies that $st\text{-lim}\;x=-{\tilde{q}}_{\sigma }\left(-Ax\right)={\tilde{q}}_{\sigma }\left(Ax\right)=\sigma_{C}\text{-lim}\left(Ax\right)$; that is, $A\in {\left(st\cap \ell_{\infty }:{\tilde{V}}_{\sigma }\right)}_{\text{reg}}$.

*Sufficiency*. Let $A\in {\left(st\cap \ell_{\infty }:{\tilde{V}}_{\sigma }\right)}_{\text{reg}}$ and let (26) holds. If *x* ∈ *ℓ*_*∞*_, then *β*(*x*) is finite. Let *E* be a subset of *ℕ* defined by *E* = {*i* : *x*_*i*_ > *β*(*x*) + *ε*} for a given *ε* > 0. Then, it is obvious that *δ*(*E*) = 0 and *x*_*i*_ ≤ *β*(*x*) + *ε* if *i* ∉ *E*. For any real number *λ*, we write *λ* ^+^ = max⁡{*λ*, 0} and *λ* ^−^ = max⁡{−*λ*, 0} whence |*λ* | = *λ* ^+^ + *λ* ^−^, *λ* = *λ* ^+^ − *λ* ^−^ and |*λ* | −*λ* = 2*λ* ^−^. Now, we can write

(43) ∑icni(p)xi=∑i<i0cni(p)xi+∑i≥i0cni(p)xi=∑i<i0cni(p)xi+∑i≥i0cni+(p)xi−∑i≥i0cni−(p)xi≤||x||∑i<i0|cni(p)|+∑i≥i0i∉Ecni+(p)xi +∑i≥i0i∈Ecni+(p)xi+||x||∑i≥i0[|cni(p)|−cni(p)]≤||x||∑i<i0|cni(p)| +[β(x)+ε]∑i≥i0i∉E|cni(p)|+||x||∑i≥i0i∈E|cni(p)| +||x||∑i≥i0[|cni(p)|−cni(p)].

Applying the operator limsup⁡_*n*→*∞*_sup⁡_*p*∈*ℕ*_, we obtained from hypothesis that ${\tilde{q}}_{\sigma }\left(Ax\right)\leq \beta \left(x\right)+\varepsilon$. This completes the proof since *ε* is arbitrary.

In the special case *σ*(*p*) = *p* + 1, we also have the following theorem.

**Theorem 25 (see [6])**

Consider that *B*_*C*_-*core*(*Ax*)⊆*st*-*core*(*x*) for all *x* ∈ *ℓ*_*∞*_ if and only if $A\in {\left(S\cap \ell_{\infty }:\tilde{f}\right)}_{reg}$ and (33) holds.
